# 

**Published:** 1914-03

**Authors:** 


### CONTENTS.

ORIGINAL COMMUNICATIONS—

Who Should Engage in the Practice of Surgery, by E. C. Davis, A. B., M. D., Atlanta, Ga. .......... 527

Visit of the Georgia Surgeon's Club to New Orleans, by O. H. Matthews, M. D., Atlanta, Ga. .......... 532

The Diagnosis of Scarlet Fever, by W. N. Adkins, M. D., Atlanta, Ga. .......... 535

A Plea for More Pelvimetry, by Frank Giuffrida, M. D., Atlanta, Ga. .......... 541

Fulton County Medical Society. Regular Meeting Thurs day, February 19, 1914, by R. R. Daly, M. D. .......... 542

Important Meeting .......... 549

To Editor of Journal Record of Medicine .......... 549

EDITORIAL .......... 550

Selections and Abstracts .......... 554

Book Reviews .......... 573

